# Massage It Out: Physiological Responses to a Percussive Therapy Device Used Intra-Resistance Exercise

**DOI:** 10.3390/muscles3020015

**Published:** 2024-06-19

**Authors:** Daniel R. Greene, Jonathan J. Ruiz-Ramie, Andrew Craig-Jones

**Affiliations:** Department of Kinesiology, Augusta University, 3109 Wrightsboro Road, Augusta, GA 30909, USA; jruizramie@augusta.edu (J.J.R.-R.); acraigjones@augusta.edu (A.C.-J.)

**Keywords:** performance, massage therapy, resistance exercise, musculoskeletal manipulation

## Abstract

Percussive therapy devices have been proven effective at reducing muscle inflammation, soreness, and tension and improving the range of motion before or after exercise. However, there is a notable lack of research on percussive therapy intra-exercise. Purpose: Examine the physiological responses (i.e., sets/reps) to percussive therapy during single-arm biceps curls (BCs) and single-leg quadriceps extensions (LEs). Methods: Participants [N = 26, 10 females] completed an initial 10-repetition maximum (10 RM) for BCs and LEs. Following that, participants completed two testing sessions in which BCs and LEs were completed at their 10 RM until functional failure (i.e., <7 reps completed). Participants completed two testing sessions in which all body parts received percussive therapy at 25 Hz for 60 s between sets and no percussive therapy. Results: Participants completed more sets [*p* = 0.002; Cohen’s d = 0.54] and reps [*p* = 0.005; Cohen’s d = 0.51] during the control condition relative to the percussive therapy condition. There were no differences between body parts (i.e., BC vs. LE) or interaction effects. Conclusion: This study provides evidence that low-frequency long-duration percussive therapy used intra-resistance exercise decreases performance parameters.

## 1. Introduction

Modern percussive therapy may owe many of its original roots to manual percussion or tapotement-based therapeutic massage. These techniques involve a practitioner applying light rhythmic striking to the skin with the ulnar portion of the hand or with hands in a cupped position [1]. A derivative of these treatments, mechanical percussive therapy, was originally developed as an osteopathic treatment for musculoskeletal pain in the 1950s by Dr Robert Fulford, administered via a percussion vibrator [2]. Decades later, in 2008, the first commercially available percussive therapy device came to market, providing increased consumer access to what was once considered a more specialized treatment [3]. Subsequently, many different products with multiple mechanical variations have come to market. With the market saturated with different products, there is a need for additional research to explore these products’ potential benefits and downsides of use.

Since the introduction of the commercially available device, percussive therapy has gained massive popularity in the sports performance realm. Percussive therapy devices are now being used both personally and professionally by therapists, strength and conditioning coaches, trainers, and athletes as a means of potential performance benefit [4]. A recent analysis concluded that percussive therapy devices have been well-received by consumers and offer an easy/affordable option for numerous health ailments including pain relief, rehabilitation, and injury prevention [5]. Nonetheless, a relatively novel potential performance aid, investigations on how to best use these devices have yet to come to a general consensus [6]. This heterogeneity in their use may be credited to differential intervention techniques, especially in the timing of therapy relative to exercise. To date, scientists have examined the effects of percussive therapy pre-, post-, and intra-exercise with varying results [7,8,9].

### 1.1. Pre-Exercise

Vibration therapy has been associated with increased performance parameters. Most notably, percussive therapy devices have been effective at improving the range of motion (ROM) and flexibility when used pre-exercise. Konrad et al. [10] found a significant improvement in maximum dorsiflexion ROM following 5 min of percussive therapy at 53 Hz relative to a control treatment. Another study showed a significant improvement in passive ROM for the hip and knee flexors as well as the ankle extensors following 30 s of percussive therapy at 40 Hz, relative to a control condition [11]. Further, eight minutes of percussive therapy at 30 Hz applied to the quadriceps was effective at increasing hip flexion range of motion in young healthy adults [12]. Also, Skinner et al. [13] demonstrated that percussive therapy applied pre-exercise can have a large, positive effect on tissue dynamics. Following two applications of percussive therapy at 29 Hz for 60 s, participants reported an increase in ROM, tone, and relaxation time, while simultaneously decreasing hamstring muscle stiffness. With ROM and tissue mechanics improving immediately after percussive therapy, there is a large potential for these devices to be utilized for injury prevention as well as ergogenic aids in an athletic environment.

While percussive therapy has shown significant benefits associated with ROM and flexibility, other performance variables have shown mixed results. A recent systematic review reported that percussive therapy devices caused no change in, or a slight decrease in, the performance variables of strength, acceleration, and agility [7]. Percussive therapy devices used before exercise have been shown to generate no difference in the maximum voluntary contraction torque of the plantar flexor muscles relative to a control condition [10]. Furthermore, Armstrong et al. randomly assigned 90 participants to a control group or a 1 min bout of whole-body vibration before completing a series of vertical jump tests. Their results indicated no significant condition effects [14]. However, significant performance improvements have been shown with percussive therapy devices used pre-exercise. A recent study noted a significant increase in ROM, isokinetic peak torque, and dynamic balance following a warm-up activity incorporating vibration rolling [15]. Although mixed, the results from the literature on percussive therapy use pre-exercise suggest a benefit to ROM and flexibility without any benefit or harm to other performance variables.

### 1.2. Post-Exercise

While the evidence is limited, some studies have assessed how the use of percussive therapy devices after exercise impacts certain performance parameters. Similar to pre-exercise, percussive therapy devices have mixed results when used post-exercise. A recent review highlights the benefit of reduced delayed-onset muscle soreness (DOMS) when percussive therapy devices are used post-exercise [9]. Primary research articles have found significant improvements in pain following the use of vibration therapy and percussive massage. Cochrane assessed muscle pain following 15 min of vibration therapy or a control condition immediately, 24, 48, and 72 h after eccentric resistance exercise. Their results indicated a significant decrease in pain at 24 (d = 0.64) and 72 (d = 0.85) hours post-exercise in the vibration therapy condition relative to the control [16]. Another study used an 11-point rating scale to compare muscle soreness following 60 eccentric elbow flexion movements in healthy college students. Using a between-subjects design, participants received one minute of percussive massage immediately, 24, 48, and 72 h following exercise or no percussive massage. Participants who received percussive massage reported significantly less muscle soreness (i.e., by ~2–3 points) at between 24 and 72 h relative to the control condition [17]. Furthermore, Leabeater et al. examined the effects of a percussive therapy device, used on one leg for 5 min at 53 Hz following a strenuous calf exercise, relative to the other leg that did not receive percussive therapy. Their results indicated no significant differences between the two conditions on ROM, isometric strength, or muscle endurance [18]. Another study assessed the ROM, perceived muscle soreness, and isometric torque following eccentric exercise. Participants received either 1 min of percussive therapy at 40 Hz or quiet rest immediately, 24, 48, and 72 h following eccentric resistance exercise. Their results highlighted a nonsignificant benefit on perceived muscle soreness and a significant benefit on ROM in the percussive therapy group relative to the control, with no differences in isometric torque [17]. Percussive therapy post-exercise may decrease DOMS; however, the effects on other performance variables appear minimal.

### 1.3. Intra-Exercise

The invention of percussive therapy devices opens up an entirely new way to assess how percussive therapy influences performance. While, in the past, percussive therapy techniques could be utilized before or after exercise, these devices can now be conveniently used during exercise. A recent study examined the effects of percussive therapy on movement velocity and muscular endurance during bench press exercise. Participants randomized into the percussive therapy group completed a significantly greater number of repetitions relative to participants in the control group, with no differences in movement velocity [8]. To the best of our knowledge, this is among the first studies to assess changes in performance intra-exercise with the use of a percussive therapy device. As such, further studies are needed to more fully explore performance changes that may be associated with percussive therapy intra-exercise. Given that the above study used percussive therapy at 40 Hertz for a relatively short duration (i.e., 15 s) [8], it seems logical to explore the effects of using percussive therapy devices at lower frequencies for longer durations. This novel study also evaluated pectoral percussion during a bench press exercise procedure. Although the pectoralis major is a large contributor to bench press work, variations in hand placement and bar trajectory may alter the participant’s work distribution to the muscles [19,20]. With little known to date about percussive therapy used intra-exercise, exploring its application during more controlled, simple lever joint exercises such as biceps curls and knee extensions may be beneficial.

### 1.4. Percussive Therapy Frequency and Duration

As mentioned above, there are numerous methodological differences in the body of literature surrounding percussive therapy use. As percussive therapy devices are mechanical, the device settings (e.g., frequency) and usage (e.g., duration) can be manipulated to explore numerous effects. Garcia-Sillero et al. [8] found an increase in the number of bench press repetitions performed while using percussive therapy during rest periods, at 40 Hz for 15 s. While there are limited studies assessing the use of percussive therapy devices during rest periods of repeated bouts of resistance exercise, numerous studies have assessed performance variables immediately following one-time use of percussive therapy. Following 30 s of percussive therapy at 60 Hz, participants reported an increase in the reactive strength index [21]. Conversely, Szymczyk et al. [22] found a decrease in athletic performance (i.e., jump height) following 60 s of percussive therapy use at 20 Hz. No difference in countermovement jump performance or drop jump landing was reported following 30 s of percussive therapy (10 s on plantar muscles) at 40 Hz in each of the major muscles involved (quadriceps, hamstrings, gluteus maximus, gluteus medius, calves, peroneals, and plantar muscle groups) [23]. While there is a clear need to establish the most optimal settings to increase performance variables following the use of percussive therapy, the results of these studies appear to support higher-frequency percussive therapy over a shorter duration to increase performance variables. This would support the tonic vibration reflex theory [24]. This theory highlights the notion that high-frequency vibration delivered over a short duration may enhance muscular performance, while low-frequency stimulation over a longer duration may have the opposite effect.

### 1.5. Purpose

Given the above information on percussive therapy devices used before, during, and after exercise, it is clear that more research needs to be conducted to determine their overall effectiveness. The purpose of the present study was to examine performance variables during exercise in conjunction with the use of percussive therapy devices at a low frequency (i.e., 25 Hz) for a longer duration (i.e., 60 s). Specifically, the maximal number of sets and repetitions performed was examined during single-arm biceps curls and single-leg extensions with and without percussive therapy applied during rest periods. Although the previous literature supports a beneficial effect of percussive therapy used intra-exercise, based on the tonic vibration reflex theory, we hypothesized that participants in the present study would complete significantly fewer sets and repetitions while using percussive therapy intra-exercise relative to the control condition. Additionally, we hypothesized that there would be no difference between body parts. Specifically, there would be no differences in biceps curls (small muscle group) relative to quadriceps extensions (larger muscle group). These hypotheses were based on the tonic vibration reflex theory [24], stating that vibration at a low frequency for long durations may hinder muscular performance while vibrations at a higher frequency for shorter durations may enhance it.

## 2. Materials and Methods

### 2.1. Participants

**Figure 1:**
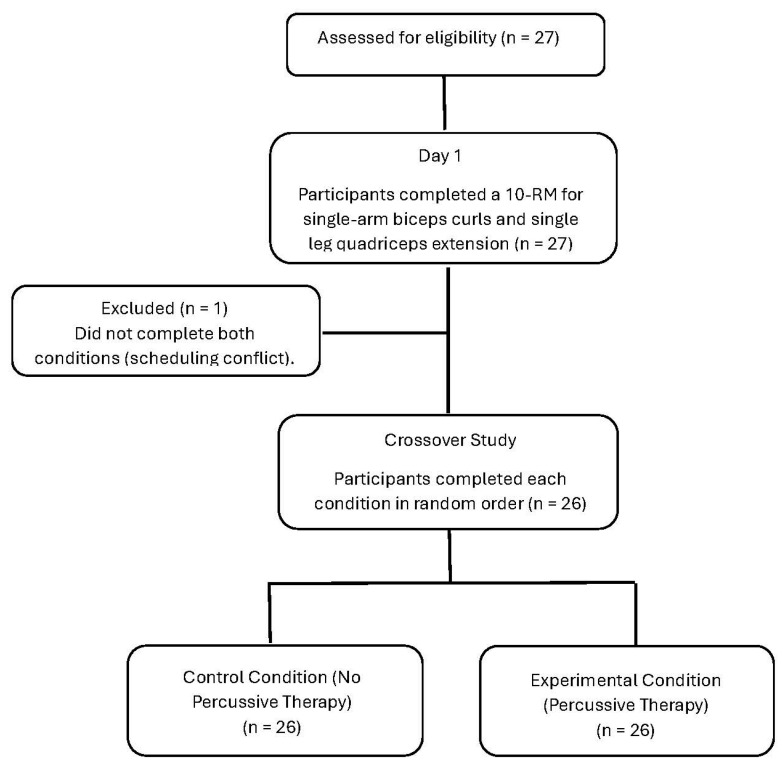
Flow diagram of participant recruitment and randomization.

Male (n = 16; 21.2 ± 2.8 years; 180.3 ± 10.1 cm; 80.4 ± 12.6 kg; 24.7 ± 2.8 kg/m^2^) and female (n = 10; 20.8 ± 1.5 years; 163.1 ± 5.5 cm; 69.3 ± 14.6 kg; 26.1 ± 4.7 kg/m^2^) participants were recruited through the Kinesiology program at a Southern University. Interested individuals completed the university-approved Informed Consent Document (reference number 1568711) and, to determine if participation was likely safe, completed a PAR-Q and health history form. Follow-up questions would have been asked if participants indicated any contraindications to exercise, though none were present. Overall, 27 participants were recruited, but the final sample consisted of 26 as one participant dropped out due to scheduling conflicts. As that participant did not complete both conditions, they were removed from all analyses (see Figure 1). Participants were highly active, with 84 percent indicating vigorous exercise participation on a regular basis. Furthermore, participants indicated exercising an average of 4.2 ± 1.1 (M ± SD) days per week over an average of 3.4 ± 2.9 (M ± SD) years, at a self-reported average exercise intensity of 5.7 ± 1.4 (M ± SD). Exercise intensity was assessed using the CR-10 RPE scale [[25] 5 = hard, 7 = very hard]. Before participation in the study, participants were asked to refrain from alcohol consumption 24 h prior to testing and to refrain from exercising 48 h prior to both testing conditions.

### 2.2. Sample Size Calculation

Using prior work on G*Power as a guide [26], a post hoc calculation was conducted to determine if the sample size was sufficient. Garcia-Sillero et al. [8] reported significantly greater performance while using a percussive therapy device during resistance exercise with an effect size of 0.867. Based on these results, the following parameters were defined using a within-subjects design: Cohen’s d: 0.867; alpha error probability: 0.05; 1-beta: 0.95. The sample size needed to detect a significant effect was determined to be 16. As such, the 26 participants that completed the study protocol were deemed sufficient.

### 2.3. Procedures

**Figure 2:**
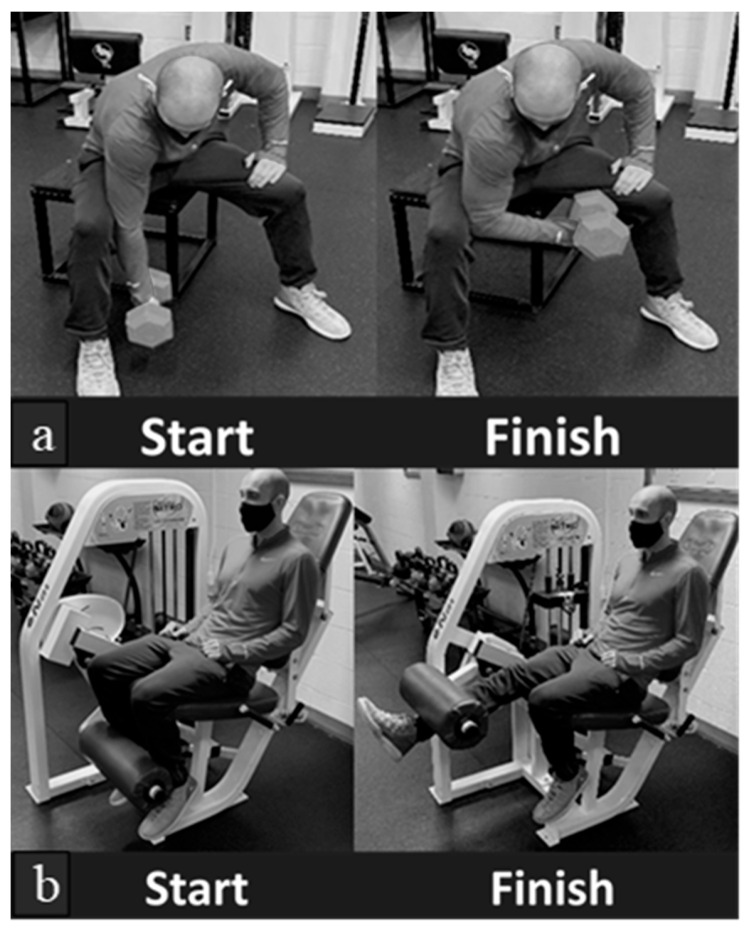
Picture of (**a**) single-arm biceps curls and (**b**) single-leg quadriceps extensions.

The present study was a randomized controlled pilot study consisting of three laboratory visits over two weeks. Following the completion of all baseline questionnaires, participants were familiarized with all testing protocols and introduced to the percussive therapy device. Also, during the first visit, participants completed a 10-repetition maximum (10 RM) for single-leg quadriceps extensions and single-arm biceps curls on both legs and arms, respectively (see Figure 2). For single-arm biceps curls, participants used a dumbbell in the position depicted in Figure 2a as “start” and finished in the position shown in 2a as “finish”. Similarly, participants used the leg extension machine depicted in Figure 2b. The 10 RM protocol was adopted from [27] and was defined as the maximum weight that could be safely lifted 10 times without sacrificing the exercise form [28]. Before assessing each participant’s 10 RM, a warm-up set was performed. Then, participants self-selected the weight they believed they could lift 10 times. For biceps curls, weight was added or removed in increments of 5 or 10 pounds depending on the level of success or failure. For example, if the participant appeared to barely complete 10 repetitions, only 5 pounds was added. For leg extensions, weight was initially added or removed in increments of 10 or 20 pounds followed by increments of 5 to 10 pounds depending on the level of success or failure. This process continued until each participant reached their 10 RM within 5 pounds for each exercise. Between attempts, participants rested for three minutes. This is the recommended rest period to ensure recovery of the phosphocreatine system used during short-duration, high-intensity activity [28]. Following the completion of a 10 RM on all 4 body parts, participants were scheduled for their first experimental condition and permitted to leave.

### 2.4. Experimental Conditions

Participants returned to the laboratory two more times to complete both experimental conditions. Participants were instructed to complete single-leg quadriceps extensions and single-arm biceps curls at their previously determined 10 RM until functional failure. Functional failure was reached when participants could not complete more than 6 repetitions (i.e., <7). During all experimental conditions, rest periods were set at 90 s. Rest periods of 1–2 min have been identified as sufficient for repeated muscular strength activities, while not permitting the individual to maintain training intensity [29]. Once functional failure was reached, participants completed the same protocol for the opposite limb until functional failure was reached for the right biceps, left biceps, right quadriceps, and left quadriceps. Conditions were randomized and counterbalanced. During the active conditions, participants self-administered the percussive therapy device during the first 60 s of each 90 s rest interval. All participants were instructed on how to use the percussive therapy device and were monitored during every testing session by no fewer than two trained research staff. Additionally, participants were required to wait 48 h between testing sessions (i.e., 10 RM, active and control conditions), asked to refrain from exercising and alcohol consumption 24 h prior to testing, completed each session around the same time of day, and completed all testing sessions within two weeks.

### 2.5. Device

**Figure 3:**
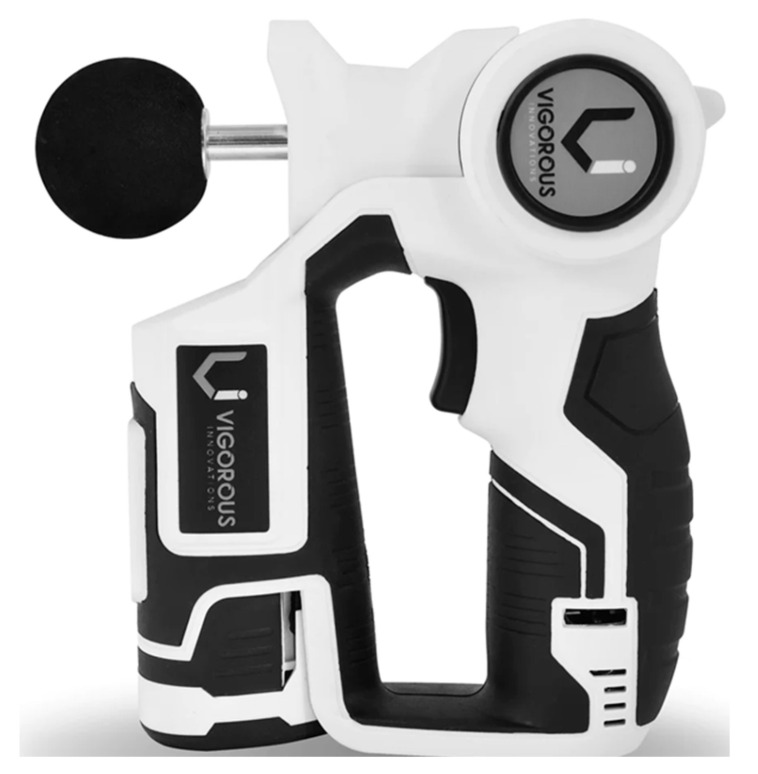
Vigorous Innovations percussive therapy device used in the present study. The device comes with three attachments: (1). Large Ball: for large core muscles; (2). Medium Ball: for small core muscles; (3). Cone: for deep tissue. The present study used the depicted “Medium Ball” for all conditions.

A high-intensity percussive therapy device from Vigorous Innovations was used during the active condition by all participants. The medium-soft ball attachment was used for both the single-arm biceps curl and the single-leg quadriceps extension exercises (see Figure 3). Given that most literature on percussive therapy devices focuses on recovery post-exercise, the present study reduced the oscillation speed from 50 Hz [9] to 25 Hz to assess the feasibility of said devices intra-exercise. The device’s amplitude was 16 mm. As per manufacturer instructions, participants were instructed to rest the percussive therapy device perpendicularly over the muscle prior to turning it on. Once engaged, participants were instructed to glide the device back and forth across the muscle or to hold the device in one spot for a maximum of 10 s. Throughout all testing conditions, participants were monitored to ensure proper use of the percussive massage device by no fewer than two trained research members.

### 2.6. Data Analysis

Statistical data analysis was conducted using SPSS 22.0 for Windows. Descriptive statistics (i.e., mean (M) and standard deviation (SD)) were computed for all participants. Data were initially inspected for any unusual data points. Analysis of differences for the numbers of sets and repetitions completed was conducted using Condition [2: Active, Control] by Exercise [2: Biceps, Quadriceps] repeated-measures analyses of variance [RM ANOVAs]. RM ANOVAs were used with a Bonferroni adjustment for multiple comparisons along with Huynh–Feldt epsilon (H-F ε) correction to protect against sphericity assumption violations. Effect sizes (ESs) were calculated at Cohen’s d [30].

## 3. Results

### 3.1. Sets

**Figure 4:**
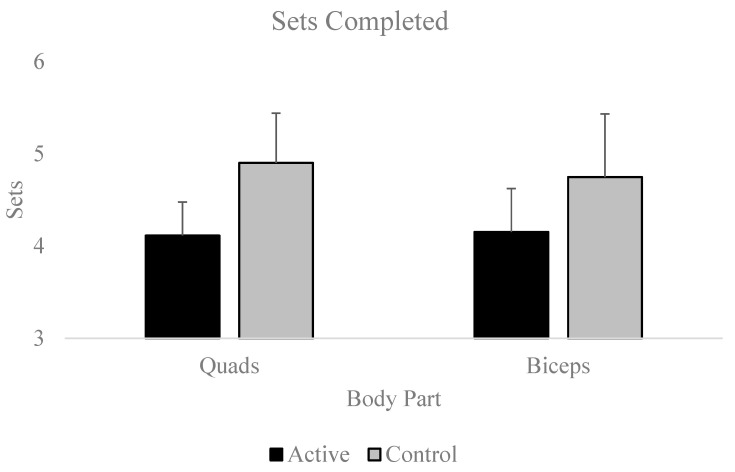
Number of sets completed, which can be compared for Condition and Exercise effects.

Repeated-measures ANOVAs [Condition (2: Active, Control) by Exercise (2: Biceps Curl, Quadriceps Extension)] revealed a significant Condition main effect [η2part = 0.327; H-F ε = 1.00], but neither the Exercise main effect nor the Condition x Exercise interaction effect were significant (ps > 0.5) for the number of sets completed. Specifically, participants completed significantly more total sets until functional failure during the control condition relative to the active condition [Mdiff ± SE; 0.69 ± 0.20; *p* = 0.002; Cohen’s d = 0.54; see Figure 4].

### 3.2. Repetitions

**Figure 5:**
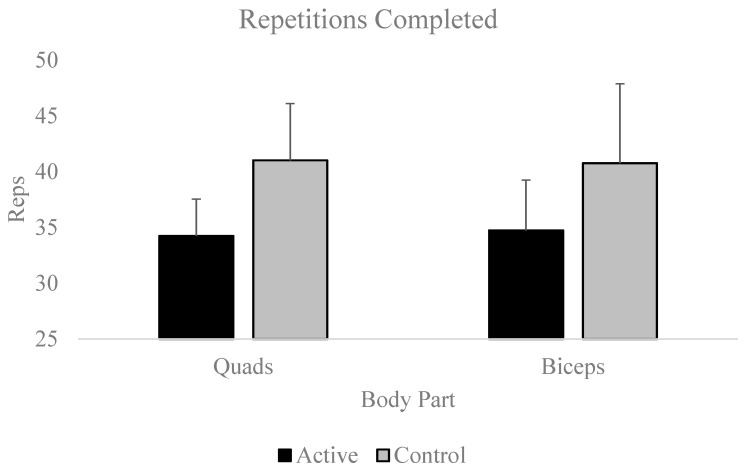
Number of repetitions completed, which can be compared for Condition and Exercise effects.

Repeated-measures ANOVAs [Condition (2: Active, Control) by Exercise (2: Biceps Curl, Quadriceps Extension)] revealed a significant Condition main effect [η2part = 0.277; H-F ε = 1.00] but neither the Exercise main effect nor the Condition x Exercise interaction effect were significant (ps > 0.79) for the number of repetitions completed. Specifically, participants completed significantly more total repetitions until functional failure during the control condition relative to the active condition [Mdiff ± SE; 6.39 ± 2.07; *p* = 0.005; Cohen’s d = 0.51; see Figure 5].

## 4. Discussion

The present study assessed performance variables during resistance exercise both with and without percussive therapy intra-exercise (i.e., during rest periods). Given the mixed literature on percussive therapy devices, further exploration of their use during exercise is warranted. While percussive therapy devices and myofascial release techniques (e.g., foam rolling) have shown promise to both increase flexibility [31] and ROM [23,32,33] when utilized before exercise as well as improve muscle recovery/pain when used post-exercise [32,34], the effects on other performance variables, specifically during exercise, are still unknown.

We hypothesized that the use of a percussive therapy device intra-exercise would decrease performance. Specifically, we hypothesized that participants would complete significantly fewer repetitions and sets of single-arm biceps curls and single-leg extensions when percussive therapy was used for 60 s at 25 Hz during the rest intervals. The results of the present study support this hypothesis since participants completed significantly fewer repetitions and sets when percussive therapy was used. Interestingly, these results are in opposition to one of the only other studies to assess these performance variables. Garcia-Sillero et al. [8] reported a significant increase in bench press repetitions when percussive therapy was used intra-exercise. While these two studies have different outcomes, some key methodological differences might advance our understanding of the use of percussive therapy devices intra-exercise. Specifically, it appears that high-frequency short-duration percussive therapy may increase performance intra-exercise, while low-frequency long-duration percussive therapy may decrease performance. Overall, the results of the present study support the tonic vibration reflex theory [24].

It was also hypothesized that there would be no differences observed with larger and smaller muscle groups. The present study had participants complete single-arm biceps curls and single-leg quadriceps extensions until functional failure. Participants completed both an active and a control condition. Overall, the results supported the hypothesis as there were no differences observed between the biceps and quadriceps muscles. While this is not surprising, it is novel in that this is the first study to assess differences between a larger and smaller muscle group. Previous studies have assessed changes in performance variables of compound movements following percussive therapy. Hernandez examined countermovement jump performance and drop jump landing following 30 s of percussive massage on all major muscles involved. The results showed no difference in either movement with or without percussive therapy [23]. Percussive therapy appears to exert the same effects on small and large muscles, but further research is needed to expand these findings to more muscles. Additionally, percussive therapy devices often come with numerous attachment heads. Konrad et al. [10] identified five attachment heads used for the Hypervolt percussive massage gun. Other attachments come with different percussive massage devices and can include round heads of various sizes (i.e., small, medium, large), flat heads designed for deep tissue massage, pointer heads to alleviate small knots, spinal heads for back muscles, and texture heads for larger muscles, to name a few. Future research should explore the use of various attachment heads for different muscles. For example, larger muscle groups may benefit from large round heads or textured heads, while smaller muscle groups might benefit from small round heads or flat heads.

To the best of our knowledge, this is the first study to assess changes in performance variables (i.e., sets/repetitions until functional failure) while using a percussive therapy device intra-exercise. The within-subjects design strengthens the overall findings and generalizability of the present study, however, there are a few limitations worth mentioning. First, the sample was a convenience sample, and previous exercise history was not controlled for. Although this was only a small concern as 84 percent of the participants indicated exercising regularly, it would be of value to explore these findings in both sedentary and highly active individuals. Additionally, the execution tempo was not specifically controlled for between repetitions. Participants observed a trained research member demonstrate the proper technique and received instruction on controlling the eccentric phase of the movement. Future research should specifically control for the execution tempo and velocity of contraction. Finally, the present study had participants complete resistance exercise conditions based on a 10-repetition maximum assessment. It is possible that the participant’s 10-repetition maximum varied over the three testing days. However, this was only a small concern as the percussive massage and control conditions were randomized. Future research should take this into account and assess changes in performance variables following chronic use of percussive therapy devices to determine if there are any long-term benefits.

## 5. Conclusions

Overall, the results of the present study support the tonic vibration reflex theory and extend this theory to apply to percussive therapy devices used intra-resistance exercise. Specifically, the results provide evidence that percussive therapy devices used intra-resistance exercise at low frequencies (i.e., 25 Hz) for longer durations (i.e., 60 s) decrease physiological performance variables. The practical implications of these findings extend to recreational exercisers, personal trainers, coaches, and anyone who incorporates a percussive therapy device into their workouts. Chiefly, we assert that the device settings and usage are vital to specific performance variables. As such, further research is needed to find the optimal settings (i.e., frequency and duration) to improve performance during exercise. In addition to the relative timing (pre-, intra-, post-exercise) and massage device settings (frequency and duration), researchers may simultaneously test various muscle groups and performance metrics within the same study. These studies would aid in uncovering potential differential responsiveness among muscle groups/performance metrics and provide a more comprehensive view of the potential benefits of mechanical percussive therapy.

## Data Availability

Data will be made available upon reasonable request from the first author.
